# Internet use by pregnant women seeking pregnancy-related information: a systematic review

**DOI:** 10.1186/s12884-016-0856-5

**Published:** 2016-03-28

**Authors:** Padaphet Sayakhot, Mary Carolan-Olah

**Affiliations:** College of Health and Biomedicine, Victoria University, St Albans Campus, Building 4C, McKechnie Street, St Albans, VIC 3021 Australia

**Keywords:** Pregnancy, Antenatal care, Information, Internet

## Abstract

**Background:**

The Internet has become one of the most popular sources of information for health consumers and pregnant women are no exception. The primary objective of this review was to investigate the ways in which pregnant women used the Internet to retrieve pregnancy-related information.

**Methods:**

We conducted a systematic review to answer this question. In November 2014, electronic databases: Scopus, Medline, PreMEDLINE, EMBASE, CINAHL and PubMed were searched for papers with the terms “Internet”; “pregnancy”; “health information seeking”, in the title, abstract or as keywords. Restrictions were placed on publication to within 10 years and language of publication was restricted to English. Quantitative studies were sought, that reported original research and described Internet use by pregnant women.

**Results:**

Seven publications met inclusion criteria and were included in the review. Sample size ranged from 182 – 1347 pregnant women. The majority of papers reported that women used the Internet as a source of information about pregnancy. Most women searched for information at least once a month. Fetal development and nutrition in pregnancy were the most often mentioned topics of interest. One paper included in this review found that women with higher education were three times more likely to seek advice than women with less than a high school education, and also that single and multiparous women were less likely to seek advice than married and nulliparous women. The majority of women found health information on the Internet to be reliable and useful.

**Conclusion:**

Most women did not discuss the information they retrieved from the Internet with their health providers. Thus, health providers may not be aware of potentially inaccurate information or mistaken beliefs about pregnancy, reported on the Internet. Future research is needed to address this issue of potentially unreliable information.

**Electronic supplementary material:**

The online version of this article (doi:10.1186/s12884-016-0856-5) contains supplementary material, which is available to authorized users.

## Background

In recent years, the Internet has become a very popular source of health information for pregnant women [1–5]. This situation has been driven by ease of access and there is evidence to indicate that pregnant women are more likely to search for information at particular times in pregnancy and in response to certain situations [3, 5]. For example, searching for health-related information prior to meeting with health professionals [3, 5] and after their consultations [6] is common. Although pregnancy is a natural event in a woman’s life, pregnancy care generally involves medical monitoring and prenatal testing [7], which can be anxiety provoking. Consequently, many pregnant women utilise the Internet as a source of information, and as a means to help them deal with doubts, and to navigate pregnancy-related decisions [2, 8, 9].

A nationwide survey in the US revealed that more than three quarters of childbearing women turned to the Internet for information about pregnancy and birth [10]. Widespread Internet searching is also reported in other countries and a Swedish study found that the majority of pregnant participants had used the Internet on one or more occasions to access information on pregnancy, childbirth or the expected baby. The majority (79 %) had looked for information during the previous month and the frequency of Internet searches varied from once a month to 62 times a month [11]. Although Internet searching is widely used, one of the difficulties with this medium is an inability to judge the quality and accuracy of retrieved information and many individuals searching online for health advice believe the information and advice they find, as reported in a previous British study [12]. This is a concern as health information provided on the Internet is not always reliable [4, 13, 14] or current [15]. Indeed, this lack of reliability is well recognised and a systematic meta-analysis of health website evaluations, found that the majority of evaluations (70 %) concluded that quality of information was a problem on the Internet [4]. A lack of clear guidelines for the user may contribute to this dilemma. Thus, it can be difficult for women to distinguish accurate from inaccurate sources on the Internet. A number of studies corroborate this finding and indicate that Internet users are hesitant about the reliability of health information they accessed [16, 17]. Without proper guidance, information on the Internet can be harmful, confusing and overwhelming [18]. Although Internet use during pregnancy offers an opportunity to share apprehensions and doubts with other women, it can also lead to increased and unjustifiable anxiety [19].

The aim of this review was to describe access and use of the Internet as the source of information among pregnant women. The questions addressed were: (i) whether, and how often, pregnant women searched the Internet; (ii) what kind of information they sought; (iii) the characteristics and stages of pregnancy of women searching the internet; and (iv) perceived reliability of the information?

## Methods

A systematic review was chosen as a suitable means of addressing the research questions. This method of establishing the current evidence base on a particular topic involves three steps: (1) a rigorous search for all relevant research papers, (2) critical appraisal of the evidence and (3) synthesis of the findings of the various papers. We used the process described by the Centre for Reviews and Dissemination [20] as this method employs a particularly rigorous review process and guides the researcher through each step.

### Search strategy, search terms, and database

In November 2014, we conducted a search of the literature on Internet use by pregnant women seeking pregnancy-related information. Only papers published in English were eligible for inclusion. This review was conducted in accordance with the Preferred Reporting Items for Systematic Reviews and Meta-Analyses (PRISMA) statement [21] (See Additional file 1 for more detail).

The search terms were as follows: “Internet” or “Internet use” or “pregnancy” or “health information seeking” or “online” or “pregnancy related-information”. Databases used in this study were: Scopus, Medline, PreMEDLINE/Ovid, EMBASE, CINAHL and PubMed. We also searched the contents of specific journals (BioMed Central, including the Journal of Pregnancy and Childbirth) and the reference lists of already retrieved papers. The author also conducted general Internet searches using combinations of the search terms in the Google search engine.

There were restrictions placed on publication to within 10 years. Papers that were published from November 22, 2004 to November 21, 2014 were eligible for inclusion. We imposed the time limitation to ensure the findings related to current practice regarding Internet using during pregnancy.

### Selection: inclusion and exclusion criteria

Inclusion and exclusion criteria were established in advance in a written protocol. The criteria for inclusion in this review were as follows:

- ▪ Papers reporting original research.
- ▪ Papers focusing on the Internet use by pregnant women.
- ▪ Papers were included when: (1) their participants were pregnant women; (2) their participants searched the Internet for health information or pregnancy related information.;
- ▪ Papers presented their results in quantitative results, such as proportions, percentages or frequencies; and
- ▪ Papers were reported in English.

Studies were excluded if they presented qualitative results; or were reviews; editorials; conferences papers and studies that dealt with content other than health information. Qualitative papers were excluded as this review was focused on understanding characteristics and patterns of Internet use by pregnant women, rather than qualitative experience.

### Selection of studies

**Fig. 1:**
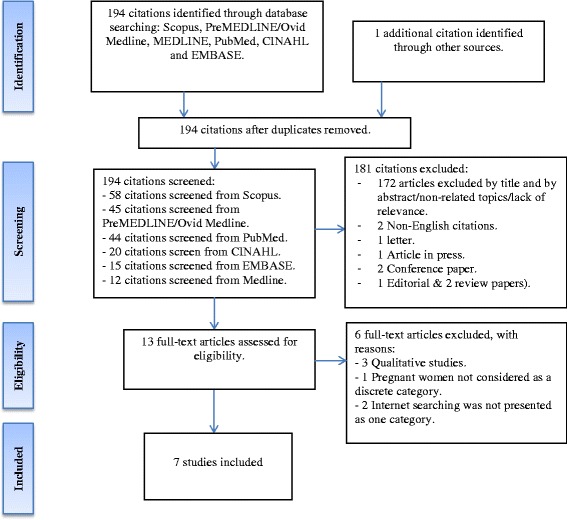
Selection of papers in accordance with PRISMA 2009 flow diagram [15]

Both authors (PS; MCO) assessed the eligibility of papers identified by the search strategy. The selection criteria was applied to titles and abstracts, with decisions erring on the side of caution, that is, to include all papers potentially reporting Internet use by pregnant women seeking pregnancy-related information. We retrieved the full text of papers assessed as potentially relevant, and both authors assessed them for inclusion. Disagreements about inclusion or exclusion of particular studies were resolved by discussion between the two authors. Studies that appeared to fulfil the inclusion criteria but were later excluded are detailed in a ‘Characteristics of excluded studies’ table (Table 2). Following this process, seven articles met the inclusion criteria [1, 5, 11, 22–25] (See Fig. 1).

### Quality appraisal/risk of bias

Both authors assessed all included papers using the scale for quantitative studies developed by Kmet et al. [26]. Using this guide, the authors assigned a score of between zero and two for each of up to fourteen aspects of each paper, including: (i) Question/objective of study sufficiently described?; (ii) Study design evident and appropriate?; (iii) Method of study/study procedure described and appropriate?; (iv) Subject characteristics sufficiently described?; (v) If interventional or random allocation was possible, was it described?; (vi) If interventional and blinding of investigators was possible, was it reported?; (vii) If interventional and blinding of subjects was possible, was it reported? (viii) Outcome well defined/means of assessment reported? (ix) Sample size appropriate? (x) Analytic methods described/appropriate? (xi) Some estimate of variance is reported for main results? (xii) Controlled for confounding? (xiii) Results reported in sufficient detail? (xiv) Conclusions supported by the results?

We then converted the number score to a percentage score. Scores ranged from 70 to 100 %, we therefore considered all papers were of sufficient quality to be included in the review.

### Data extraction and analysis

Data were extracted from all included studies using electronic data extraction forms. The data extraction form was pilot tested with the first five included studies and refined as was considered necessary. The following data were extracted:

- Title, author, date of study;
- Details of the study including aim, design, participant recruitment/inclusion, research ethics, use of appropriate statistical methods;
- Assessment of study quality;
- Category of Internet health seeker, location, setting;
- Results of study.
- Limitations of study.

Extracted data were managed in an endnote library and were presented in a table (Table 1). The authors did not intend to conduct a meta-analysis of the results due to the differences of the papers in terms of population and data collection. Analysis of the findings involved identifying key topics reported in each paper as outlined in the Centre for Reviews and Dissemination Guidance for Undertaking Reviews in Health Care [20]. The findings of this review are presented below.

**Table 1:** Summary of papers included in the review

Authors and title	Ami(s)	Method	Participant	Main results or findings	Kmet et al. (2004) [26] score and quality issues
Bakhireva LN, et al. (2011) [22]	To examine information sources about the safety of medications during pregnancy among pregnant women.	• Tool: Questionnaires • Location: University of New Mexico clinics • Recruitment: via a bilingual interviewer.	404 Latina pregnant women.	Internet, books and brochures were the most frequent self-identified sources of information. Women with higher education were three times more likely to seek advice than women with less than a high school education. Married and nulliparous women were more likely to seek advice than single and multiparous women, respectively.	Score = 15/20 = 75 % Study design, method and results are not sufficiently detail. No Estimating of variance reported for the main result.
Bert, F. et al. (2013) [23]	To estimate the prevalence of pregnancy e-health seekers in a large Italian sample.	• Tool: Questionnaires • Location: Multicentre (7 Italian cities: Cassino, Chieti, Palermo, Roma, Siena, Torino and Udine). • Recruitment: via medical doctors at outpatient waiting rooms from 2011–2012.	1347 pregnant women, aged 18–44 years.	The majority of women (95 %) were pregnancy e-health seekers. Most women search for information on the Internet once a month or more, most often during early stages of pregnancy. Main reason for searching the web was the need of further knowledge on pregnancy-related topic.	Score = 20/20 = 100 %
Gao, L.L. et al. (2013) [5]	To investigate whether and how Chinese pregnant women used the Internet to retrieve pregnancy-related information.	• Tool: Questionnaires • Location: Antenatal clinic at Guangzhou hospital, China. • Recruitment: via a waiting-room at the antenatal clinic from September to October in 2011.	335 Chinese pregnant women at least 32 weeks.	The majority of the women (91.9 %) had access to the Internet. Most women (88.7 %) had used the Internet on one or more occasions to access information on pregnancy, childbirth or the expected baby. The frequency of Internet searches varied from once a month to 30 times a month.	Score = 17/20 = 85 % Method not appropriate.
Huberty J., et al. (2013) [25]	To determine how pregnant women use the Internet for health information during pregnancy including information related to physical activity and nutrition.	• Tool: Online survey and paper questionnaires were used. • Location: 1). Web study based in USA, 2). Women Infant and Children clinics, family physicians, hospital prenatal courses. • Recruitment: via handouts provided in person, and via local websites from March and December 2011.	293 women, who were currently pregnant or up to 1 year postpartum.	Almost all women (94 %) reported using the Internet for pregnancy related information. Women reported using the Internet six to ten times for general health information about their pregnancy. Half of the women used the Internet for information related to physical activity during their pregnancy and some increased their physical activity as a result.	Score = 18/20 = 90 % Study design and method partially appropriate.
Kavlak O, et al. (2012) [24]	To determine the extent to which pregnant women obtain information from the Internet concerning their pregnancy.	• Tool: Questionnaire • Location: Two hospitals in Izmir, Turkey (Gynaecology and Maternity Hospital and Ege University Faculty of Medicine Hospital). • Recruitment: via outpatient antenatal clinic between August and October 2009.	185 Pregnant women in at least the 28th week of pregnancy.	44.1 % of pregnant women had used the Internet to obtain information during their pregnancy from one to two times a week. The stages of birth (92.8 %), fetal development (81 %) and nutrition in pregnancy (58.3 %) were the most researched topics. There is a significant difference between the age group, educational level, work status and number of pregnancies and the usage of Internet among pregnant women (p ≤ 0.05).	Score = 14/20 = 70 % Question, study design, sample and estimate of variance are not adequate.
Larsson, M. (2009) [11]	To investigate whether pregnant women used the Internet to retrieve pregnancy-related information, how they perceived the reliability of the information, and whether they discussed this information with their health providers.	• Tool: Questionnaire • Location: 11 antenatal clinics in a county in mid-Sweden. • Recruitment: via waiting-room from 11 antenatal clinics in a county in mid-Sweden during two weeks in 2004.	182 Swedish pregnant women (mean age = 31 years), who were at least 32 weeks pregnant.	91 % of the women had access to the Internet and, 84 % used it to retrieve information, most often in the early stages of their pregnancy. The frequency of Internet searches varied from once a month to 62 times a month. Fetal development and stages of childbirth were the two most often searched topics. Most participants considered the information to be reliable.	Score = 14/20 = 70 % Study design and subjects is limited.
Lagan, B. M. et al. (2010) [1]	To ascertain why and how pregnant women use the Internet as a health information source, and the overall effect it had on their decision making.	• Tool: Online survey • Location: Web study based in UK. The questionnaire was uploaded onto the University of Ulster server, and 23 website moderators agreed for the study to be promoted on their specific site. • Recruitment: via Internet advertisements between July and September 2006.	613 women who were pregnant or had a baby in the last year. Women were from 24 countries.	Most women (97 %) used search engines such as Google to identify online web pages to access a large variety of pregnancy-related information. All women (97 %) reported going online at least once to search for information on pregnancy products and two-thirds (67.4 %) to seek a second opinion. The majority of women (83 %) used Internet to influence their pregnancy decision-making. Statistically, women’s confidence levels significantly increased with respect to making decisions about their pregnancy after Internet usage (*p* < 0.05).	Score = 14/20 = 70 % Question, study design, method of subject and subject are not sufficiently described and appropriate.

## Results

### Study selection outcome

Using the above search terms, the author retrieved 58 citations in Scopus, 45 citations in Ovid Medline/PreMEDLINE, 12 citations in Medline, 15 citations in EMBASE, 20 citations in CINAHL and 44 citations in PubMed. In total, the author screened 194 citations. These abstracts were screened for potential relevance, including study characteristics; recruitment and participants; and quality assessment.

### Study characteristics

Seven studies met inclusion criteria and were included in the review. All seven studies aimed to find out how often pregnant women searched the Internet, what kind of information women looked for, and how they perceived the reliability of the information. Five out of seven studies [5, 11, 22–24] used paper questionnaires, two studies [1, 25] used online surveys. The characteristics of each study are presented in Table 1.

Six out of thirteen potential papers on Internet and pregnancy were excluded at full-text screening due to (a) a failure to deal with pregnant women as a discreet category [27–31], and (b) Internet searching was not a key focus of the study, or was not considered as a discreet category [9, 27–31]. A full list of excluded studies, along with reasons for exclusion is presented in Table 2.

**Table 2:** Characteristics of excluded studies (*ordered by study ID*)

Study	Reason for exclusion
Hämeen-Anttila et al. (2013) [27]	The study presented a wide variety of information sources that pregnant women used during their pregnancies and Internet searching was not presented as one category.
Laz & Berenson (2013) [28]	The study recruited all women (pregnant/non-pregnant) who searched the Internet for a variety of health problems, including menstruation, contraception, pregnancy, sexually transmitted infections. Data in relation to pregnancy information were not considered separately.
Rodger et al. (2013) [29]	The study reported on a wide variety of social media and information and communications technologies, including: email, texting, Internet, websites, YouTube, and smartphone applications that women used during pregnancy. Internet searching was not presented as one category.
Shieh, Mays, McDaniel, & Yu (2009) [30]	The study focused on health literacy and its association with the use of information sources including the Internet. Barriers to information seeking were also discussed. Data collection was not focussed on internet use in pregnancy.
Song et al. (2012) [9]	Qualitative study. This article explored the way in which women used the Internet to manage their pregnancies and mediated their doctor–patient relationships. A particular emphasis was on the role of social class and personal health history in shaping Internet use.
Weston & Anderson, 2014 [31]	Qualitative study. This study recruited three distinct groups of women: midwives, pregnant women and postnatal women. The study did not only focus on pregnancy women.

### Recruitment and participants

Studies were conducted in a variety of countries, including: the UK [1], China [5], Sweden [11], Mexico [22], Italy [23], Turkey [24], and USA [25]. Two studies recruited participants from web-based surveys in the UK and the USA [1, 25]. Five studies recruited participants from waiting rooms of outpatient antenatal clinics at multiple hospitals [5, 11, 22–24]. Participants in each study were currently pregnant or up to 1 year postpartum. In total, this review includes 3,359 participants (range from 182–1347 participants in each study). The majority of participants were pregnant women who used the Internet as a tool for searching pregnancy related-information.

### Quality assessment/Risk of bias within included studies

One study was assessed as very good quality and was scored 20/20 = 100 % [23], two studies were assessed as good quality and were scored 18/2 = 90 % [25] and 17/20 = 85 % [5], the fourth study was scored 15/20 = 75 % [22], and the final three studies were scored 14/20 = 70 % [1, 11, 24]. Quality assessments are shown in Table 1.

### Findings

Results of the data synthesis fell under four main themes, including (1) Characteristics of women who searched the Internet, (2) Frequency of Internet searching, (3) Types of information sought, and (4) Women’s perceptions of the reliability and usefulness of retrieved health information. These four main themes are described below.

### Characteristics of women who searched the Internet

All seven papers reported that the majority of women selected the Internet as a source of information about their pregnancy [1, 5, 11, 22–25]. Two papers reported that most women had access to the Internet (91.9 % and 91 %, respectively) [5, 11]. One paper found that education, marital status and parity were important predictors of information-seeking behavior. Especially, women with higher education were three times (95 % CI 1.2–7.5) more likely to seek advice than women with less than a high school education. Single (OR = 0.3; 95 % CI 0.1–0.7) and multiparous (OR = 0.4; 95 % CI 0.1–0.9) women were less likely to seek information than married and nulliparous women, respectively [22]. There were significant differences between the age group, educational level, work status, number of pregnancies and the usage of Internet among pregnant women (*p* < 0.05). Women aged 25–34 years reported using the Internet more frequently than women aged 18–24 years and ≥ 35 years old (*p* ≤0.01). Moreover, women who are employed reported using Internet more frequently than unemployed women (*p* ≤ 0.001) and first time pregnant women reported more frequently access to the Internet than multiparous women (*P* ≤ 0.01) [24].

### Frequency of internet searching

The number of times women reported using the Internet for specific reasons varied widely. All papers reported that women searched information on the Internet at least once a month or more [1, 5, 11, 22–25]. Bert et al. [23] found that women most often searched for information on the Internet during the early stages of pregnancy and these authors suggested that this finding was related to their new life situation. Kavlak et al. [24] reported that 44.1 % of pregnant women had used the Internet to obtain information during their pregnancy, at a frequency of one to two times a week [24]. Larsson [11]’s study reported a much wider frequency of Internet searches, which varied from once a month to 62 times a month.

### Type of information sought

The most often mentioned topics of interest included fetal development, nutrition in pregnancy, medications in pregnancy, pregnancy complication and antenatal care [5, 11, 22–24]. Kavlak et al. [24] study found that the majority of pregnant women (92.8 %) reported the stages of birth as the first most searched topic, following by fetal development (81 %) and nutrition in pregnancy (58.3 %), respectively [24]. Larsson [11], Gao et al. [5] and Bert et al. [23] all found that pregnant women were intensely interested in fetal development [5, 11, 23]. Gao et al. [5] also found that women sought information on nutrition during pregnancy and Bert et al. [23] additionally reported that pregnant women also desired information on prenatal tests calendars. Meanwhile, Bakhireva et al. [22] study reported that questions about the safety of medications in pregnancy were common among pregnant Internet users (62.1 %).

### Women’s perceptions of the reliability and usefulness of retrieved health information

Four studies [1, 11, 23, 25] reported women’s perceptions of health information on the Internet as reliable and useful. Lagan et al. [1] study reported that the great majority of women (96.2 %) perceived the information they located on the Internet to be “useful”. This study also found that women’s confidence levels significantly increased with respect to making decisions about their pregnancy after Internet usage (*p* < 0.05). Bert et al. [23] study reported that the main reason for searching the web was the need of further knowledge on pregnancy-related topics. This was rated more important than other key advantages of the net, such as anonymity, simplicity and rapidity. This study also reported women who searched institutional websites, declared more confidence in the information retrieved, and participated in pregnancy-centred forums online [23]. One recent study by Huberty and co-workers [25] reported that half of the women who used the Internet searched for information related to physical activity during their pregnancy and some increased their physical activity as a result. Women also reported an increase in their confidence in making decisions related to physical activity during pregnancy, after using the Internet.

Larsson et al. [11] reported the two most important criteria for judging the trustworthiness of web-based information were consistency with information from other sources, and the presence of references. They concluded that the majority of women (70 %) did not discuss the information they had retrieved from the Internet with their midwife, but more than half (55 %) sought consistency of information when they searched on topics brought up by the midwife. Although most studies indicated the converse, Kavlak et al. [24] study reported that 51 % of pregnant women stated that they shared the information, which they had obtained on the Internet with health professionals.

## Discussion

Pregnant women are very likely to search for health information online and the Internet plays an important role in providing and supporting women with health information during pregnancy. However, there is little evidence to indicate the quality of information accessed. This review describes access and use of the Internet among pregnant women and aimed to discover how often pregnant women searched the Internet, the type of information they sought and their views of the reliability of the information. These aims have been achieved and the main findings of this review indicate that most women had access to the Internet and used it to retrieve information about pregnancy, childbirth and the expected child. These three topics are the most frequently accessed by pregnant women. Most women considered the information they found to be reliable and useful and did not discuss information they had retrieved from the Internet with their physicians or midwives. This is consistent with a previous study by Diaz et al. [32], which also revealed that patients did not discuss information retrieved from the Internet with their caregivers, unless the health-care worker initiated the discussion.

Women access the Internet during pregnancy because of an “information need”, although study indicate that physicians provide them with information about pregnancy during their visit at the clinic [32], However, women still require additional information about pregnancy to improve their confidence, and used the Internet as a source of information before a prenatal visit or immediately after a visit [25]. This evidence is consistent with our review, as all seven papers reported that the majority of women searched the Internet in this way [1, 5, 11, 22–25]. De Santis et al. [19] study concurred that using Internet is the easiest and fastest way to become informed and to ease concerns. The finding highlights an important point for clinical practice as it may assist with identifying women who are most likely to search the Internet. This information may in turn, assist with development of strategies to equip to distinguish accurate versus inaccurate information and to promote healthy outcomes.

The Internet has a significant impact on everyday life [33]. Most women in this review had searched for information on the Internet at least once a month [1, 5, 11, 22–25], and this is consistent with national trends of Internet use to obtain health related-information [34]. Women in this review reported that they most often used the Internet during the early stages of pregnancy [11, 23] and this finding is similar to an Italian study by De Santis et al. [19] who found that 72 % of the women consulted a web source in the first trimester of pregnancy. Correspondingly, Hildingsson et al. [35] study on women’s expectations of antenatal care found that information about early pregnancy and fetal development was keenly sought by women. This finding suggests that women may need information early in pregnancy about fetal development, which will lead to improved confidence and have an impact on decision making. Lagan et al. [1] concurred with this finding and indicated that almost all women (83 %) used the Internet to influence their pregnancy decision making.

A qualitative study of online information seeking among pregnant women and mothers of young children in a Southeastern US city also revealed that most women sought information on the Internet during pregnancy and women had used the Internet to search for information on fetal development and stage of pregnancy. Many women also reported that they sought social support on the web from other pregnant women or mothers, especially during their first pregnancy [17]. This finding was confirmed in this review. Similarly, Hildingsson et al. [35] who examined Swedish women’s expectations of antenatal care, found that women were intensely interested in the expected baby. This notion also presents in a European study conducted in Scotland, Switzerland and Netherlands, which demonstrated that pregnant women needed to feel confident about the development of their fetus [36]. Two papers in our review, by Larsson [11] and Kavlak et al. [24] found that pregnant women were also interested in information about nutrition during pregnancy.

Similar to earlier study [30], education level, work status and number of pregnancies exerted an influence on Internet use and access among pregnant women, in this review. This review found that women with higher education were more likely to seek advice than women with less than a high school education (*p* ≤ 0.05); employed women were more likely to access the Internet and health seek information than unemployed women (*p* ≤ 0.001), and nulliparous women were more likely to seek advice than multiparous women [22, 24]. Song et al. [3] study on information needs, health seeking behaviors, and support among low-income expectant women, found that the Internet was not widely used by pregnant women who were low-income and low education level. This may suggest that some women may not have access to the Internet or a computer, while others may not have the skills or desire to search for health information online.

This review found that the majority of pregnant women with higher education perceived the health information found on the Internet to be trustworthy, reliable and useful [1, 11, 23, 25]. Many women reported that their confidence levels significantly increased with respect to making decisions about their pregnancy after Internet usage (*p* < 0.05) [1]. Huberty and co-workers [25] study of Internet searching for information related to physical activity during their pregnancy also reported an increase in confidence in decision-making related to physical activity during pregnancy after using the Internet [25]. This finding may suggest that higher education level is linked to advance reasoning skills and critical thinking and women therefore may have considered themselves to be good judges of the reliability of the information. In contrast, a study by Shieh, C., et al. [30] on health literacy and its association with the use of Internet, argued that Internet use might not have been as different between low and high health literacy pregnant women if both groups had the same amount of access and skill training. Thus, these findings imply that educational interventions to empower low health literacy women with pregnancy-specific knowledge and with information-seeking skills may be useful.

### Limitations

This review has some limitations that deserve attention. They include possible publication bias as we only included papers from November 22, 2004 to November 21, 2014 and articles written in English. This strategy may have excluded other studies or groups that could contribute insight into Internet use among pregnant women.

In addition, by excluding qualitative studies, this review may have lost some information regarding pregnant women’s Internet use.

### Implications

This review has several relevant implications for clinical practice and future research. The demographics of Internet use indicate that pregnant women are more likely to search for information during early pregnancy. Most women searched the Internet if they were pregnant for the first time, employed, educated and aged between 24–35 years. These women did not discuss information they had retrieved from the Internet unless the health care provider initiated the discussion. It is therefore important that health professionals are aware of the information pregnant women seek. Health providers are in a position to guide pregnant women’s Internet searching, by providing reputable website information and by warning women about the confusing and inaccurate information that is widely available on the Internet. Additionally, it is important that women are advised that health information on the Internet cannot be considered as a substitute for professional information and advice, and pregnant women should be cautioned not to take any action before consulting with a health-care professional [19]. Health professionals including doctors, midwives and nurses need to be more knowledgeable about common Internet sites sourced by women. This will help them to evaluate the reliability of information [37].

## Conclusion

Pregnant women often use the Internet to retrieve information on various topics related to pregnancy, including stages of childbirth, fetal development and nutrition in pregnancy. This review found that women with a first pregnancy searched for information on the Internet in early pregnancy, and also women who were employed and with higher education were more likely to use Internet. Most women perceive Internet information to be useful and reliable. However, few women discuss information found on the Internet with health professionals. This creates a potential for women to be misinformed and perhaps made needlessly anxious about pregnancy issues. Therefore, health professionals, midwives and antenatal care providers should be aware of this issue and provide more evidence based information to these women at the time they require it. Future research should address ways to better inform women of the hazards of Internet searching.

## Additional file

Additional file 1: PRISMA 2009 Checklist*. PRISMA Checklist contains 27 checklist items relevant to the content of a systematic review, which includes the title, abstract, introduction, methods, results, discussion and funding. (DOC 58 kb)
